# Ultrasound Features Associated With Shoulder Complaints: Calcifications Larger Than 6 mm in Young Patients and Positive Doppler Are Associated With Pain

**DOI:** 10.3389/fmed.2021.715423

**Published:** 2021-11-19

**Authors:** João Janeiro, Sofia C. Barreira, Patrícia Martins, Pedro Ninitas, Jorge Campos, João E. Fonseca

**Affiliations:** ^1^Serviço de Imagiologia Geral, Hospital de Santa Maria, Centro Hospitalar Universitário Lisboa Norte, Centro Académico de Medicina de Lisboa, Lisboa, Portugal; ^2^Serviço de Reumatologia e Doenças Ósseas Metabólicas, Hospital de Santa Maria Centro Hospitalar Universitário Lisboa Norte, Lisboa, Portugal; ^3^Unidade de Investigação em Reumatologia, Instituto de Medicina Molecular, Faculdade de Medicina, Universidade de Lisboa, Centro Académico de Medicina de Lisboa, Lisboa, Portugal; ^4^Serviço de Imagiologia Neurológica, Hospital de Santa Maria, Centro Hospitalar Universitário Lisboa Norte, Centro Académico de Medicina de Lisboa, Lisboa, Portugal

**Keywords:** shoulder pain, ultrasonography, Doppler, rotator cuff, tendinopathy, calcifications

## Abstract

**Objectives:** To identify ultrasound (US) features associated with the presence of shoulder complaints.

**Methods:** This observational, case-control study, compared US findings between participants with and without shoulder complaints, matched for age, sex, and dominancy. Data was collected from February 2018 to June 2020. Two-tailed Fisher's and Mann-Whitney *U*-tests were used, with *p*-values < 0.05 considered significant.

**Results:** A total of 202 participants were enrolled (median age 56 years, range 18–70, 155 women), comprising 140 cases and 62 controls. A calcification size ≥6 mm, when age < 56 (*p* = 0.02), and a distance to tendon insertion ≥6 mm, when age ≥56 (*p* = 0.009), were only found in symptomatic shoulders. Color Doppler in rotator cuff (RC) tendons predominated in the presence of symptoms (26/140 vs. 2/62, *p* = 0.003). An algorithm also combining the number of calcifications, tendon echotexture and insertional thickening, osseous irregularity, cuff tears, and subacromial effusion showed a 92% (57/62) specificity for shoulder pain on this study sample.

**Conclusion:** Calcification diameter of 6 mm or more is associated with shoulder pain in patients younger than 56 years. A distance from calcification to tendon insertion of 6 mm or more is related to pain in older patients. Doppler signal also is associated with shoulder pain. An algorithm based on a set of specific ultrasonographic criteria have a strong association with the presence of symptoms.

## Introduction

Shoulder is one of the most common anatomical sites for musculoskeletal pain, with a higher incidence in women and reaching an annual cumulative incidence of 2.4% among adults aged 45–64 years. Calcific tendinitis and other rotator cuff (RC) disorders are the most frequently diagnosed intrinsic shoulder conditions (1–7). However, shoulder pain may be due to other intrinsic causes or referred pain.

Calcifications have long been associated with pain, but also recognized to be present in asymptomatic shoulders (8). Of interest, the size of calcification is relevant and shoulders with radiological detected calcific deposits of >1.5 cm in length have the highest chance of being symptomatic (9, 10). Calcifications are also more common in women and in subjects aged between 30 and 60 years old (10). Calcifications may be due to a degenerative process, associated with trauma and tissue hypoxia and related to aging, occupation, and the use of the dominant arm, or occur in a viable tissue due to a cell-mediated reactive process, with distinct predictable stages (11). Ultrasound (US) is considered to be reliable in the detection and localization of RC calcifications (12) and US assessment of patients with known calcifications also found larger calcified plaques in symptomatic than in asymptomatic shoulders (13). However, the evaluation of a general population based cohort (women with and without calcified deposits) could not confirm the relationship between pain and calcification size and found calcifications larger than 1 cm to be unusual (14). Thickening, echotexture changes and tears in RC tendons, subacromial bursal widening, and signs of subacromial impingement and acromioclavicular osteoarthritis have been described not only in painful but also in asymptomatic shoulders (15, 16).

Detection of a positive Doppler signal within or close to a calcification was reported to be related with pain (13, 17). The location of a calcification in the medial-lateral direction was suggested to be a prognostic factor (18), with worse outcomes of non-surgical treatment for calcifications more medial and distant from tendon insertion.

The purpose of this study was to identify US features associated with the presence of shoulder complaints.

## Materials and Methods

### Study Design and Participants

This was an observational, case-control study, intended to compare US findings between participants with and without shoulder pain. The study was approved by the Ethics Committee of our institution (Approval Letter Ref. n. 413/17). Informed consent was obtained from all individual participants included in the study. Data was collected between February 2018 and June 2020 and anonymously recorded. Some of the participants (127) were included in a previously published study addressing only clinical variables (19).

Patients suffering from shoulder pain, 18–70 years old, referred for unilateral shoulder US in our hospital radiology department were consecutively recruited, until 140 cases were included. The sample size aimed a power of 80% to detect a 35% difference in the proportion of cases between exams with positive and negative findings (20, 21). Controls were randomly selected, and invited to voluntarily participate, between individuals referred to the same radiology department for other US examinations (excluding upper limb); selection aimed to match sociodemographic characteristics, as one control was selected for each two cases already recruited with the same age (decade), sex, and laterality (pain in dominant side or contralateral).

**Figure 1:**
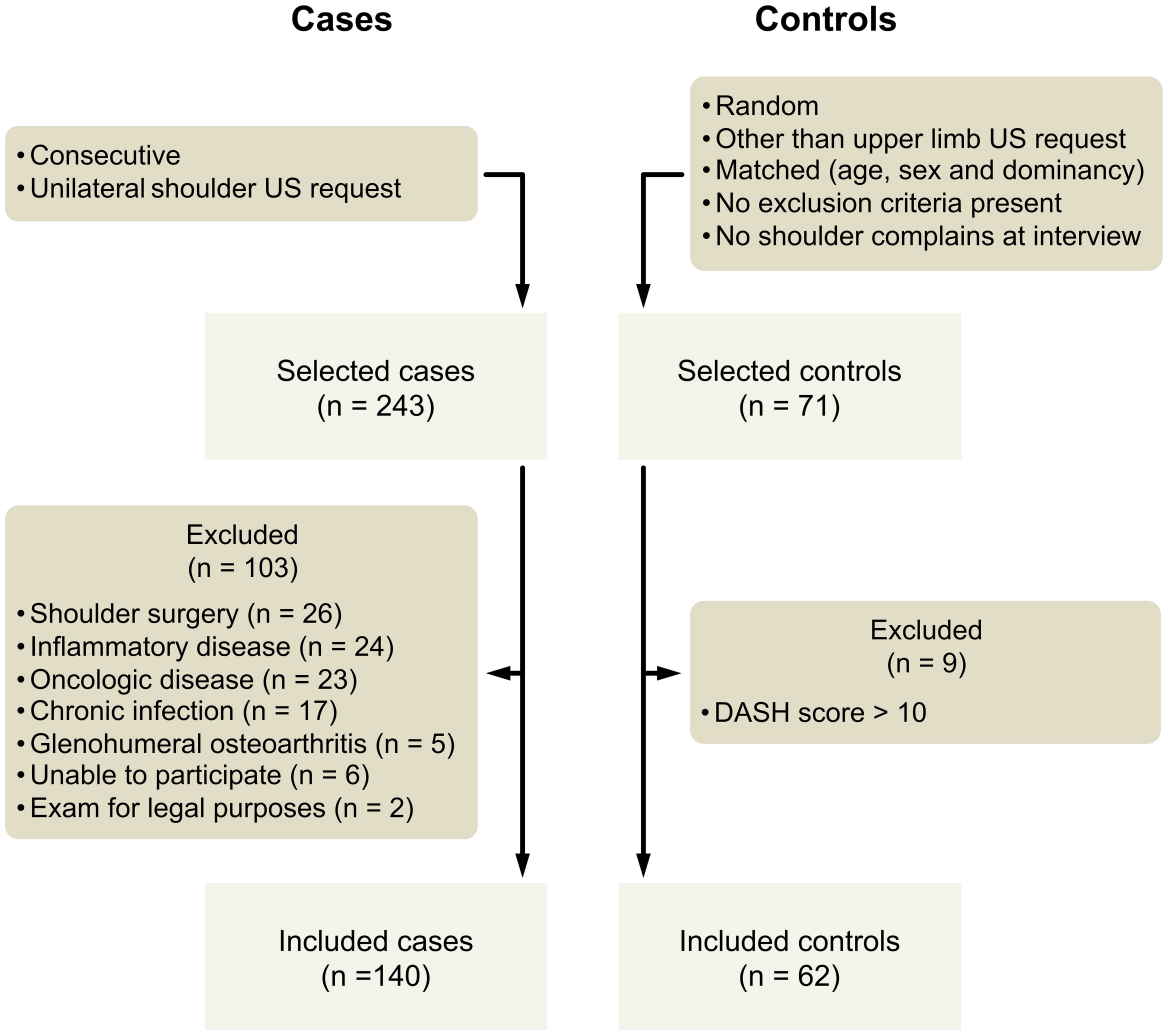
Diagram of study participants recruitment.

Exclusion criteria (Figure 1; Supplementary Material) were applied to all participants. Patients referred for bilateral shoulder examination were not selected to avoid bias. Controls were not included if they referred shoulder complaints at first interview or scored more than 10 points on a DASH (disabilities of the arm, shoulder, and hand outcome measure) questionnaire (22).

### Measurements

Participants filled a written form, including a DASH questionnaire scored as described in literature (23). Sociodemographic and clinical data were collected by interview; profession was classified according to the International Standard Classification of Occupations (ISCO 08) (24).

A physical exam was performed by one of two rheumatology residents. Active range of motion was registered, and total degrees lost calculated as the sum of the differences to maximum values (180° for abduction and flexion and 90° for internal and external rotation).

The US exam was performed to all participants by a radiologist with 16 years of shoulder US experience, blinded to the results of questionnaires and physical exam. The exam was performed with an *Acuson S2000* (Siemens Medical Solutions USA Inc., Issaquah, WA) with a multifrequency linear probe (*14L5*) operating between 5 and 14 MHz (minimum 11 MHz used). In random participants (*n* = 15) a second exam was performed by a radiology resident, for an inter-observer reliability assessment. The exam took place with the patient sitting in front of the examiner and followed described procedures (25–27). The long head of the biceps tendon was evaluated with the arm in a neutral position and the forearm on patient's lap. The subscapularis tendon was examined with the arm in external rotation. The supraspinatus tendon was examined with the patient's shoulder in internal rotation, with the hand placed on the back. The infraspinatus and teres minor tendons were evaluated with the patient's hand again on her/his lap, in slight internal rotation. All tendons were scanned on their long and short axis.

Rotator cuff calcifications were defined, as in previous studies (13, 14), as echogenic focus with or without posterior acoustic shadow, identified and measured in two orthogonal planes. The total number was registered and the six largest were described by its largest diameter, location in lateral-medial direction disclosed by the distance to osseous insertion in a direction parallel to tendon fibers, location in a particular tendon or structure, shape geometry, regularity and definition, intensity of acoustic shadow, and grouping.

Changes in tendon echotexture were reported as heterogeneity (variable echogenicity along the tendon) or hypoechogenicity (compared to muscle) and graded (mild—possible change; moderate—definite change). Tendon rupture (17, 25, 26) was classified as full or partial thickness. Subacromial conflict was assessed (28–31).

Color and Power Doppler used a pulse repetition frequency (PRF) of 488 Hz, as previously described (13, 17), with gain set at the highest gain which provided no or only minimal noise, and were graded from 0 to 3. A twinkling artifact was considered when a fast alternance between colors near the limit of spectrum was found.

### Statistical Analysis

A Fisher's exact test was used to compare frequencies of categorical variables between groups and a Mann-Whitney *U*-test was employed to compare distribution of continuous or ordinal data, except when a normal distribution was found (using Shapiro-Wilk's statistics) and an independent-samples *t*-test could be applied. Spearman's coefficient was used for bivariate correlations.

Findings with significant or near significant (*p* < 0.10) differences were further investigated in two similar size age subgroups (predefined to be below or above median age) and cutoffs derived from analysis of receiver operating characteristic (ROC) curves were applied.

Regarding reliability, Cohen's Kappa coefficient, and intraclass correlation coefficient (ICC), based on a single measurement, absolute-agreement, two-way mixed-effects model, were used. The influence of possible confounding variables differing between cases and controls was assessed by sensitivity analysis.

An algorithm was constructed which incorporated US significant variables, as major criteria if specificity in the study sample was at least 97%, and as minor criteria otherwise or if criteria was subjective (tendon echotexture changes and thickening); age subgroup or specific location criteria were applied if they provided better significance or specificity. The number of minor criteria required was defined using ROC curves analysis. Confidence intervals (CI) for sensitivity and specificity were calculated by Wald method (32).

All tests used were two-tailed and *p*-values <0.05 were considered significant.

Data was analyzed using SPSS statistical software, version 24 (IBM corp., Armonk, NY, USA).

## Results

### Characteristics of the Groups

From the 243 cases selected, 103 were excluded by predefined criteria (Figure 1). Of particular relevance, chronic inflammatory disease patients were excluded, including systemic lupus erythematosus (*n* = 10), rheumatoid arthritis (*n* = 5), ankylosing spondylitis (*n* = 2), and idiopathic inflammatory bowel disease (*n* = 2). The sociodemographic and clinical characteristics of the 202 participants (140 cases and 62 controls, median age 56 years) are displayed in Table 1. Cases and controls showed no difference in sex, age, dominancy of examined shoulder, or occupation. In the cases' group, 139 participants had shoulder pain in the previous week (one participant had only complaints of numbness), 123 reported loss of strength, 113 numbness, and 100 loss of mobility. A positive physical exam was more frequent in cases than in controls (127/140 vs. 2/62, *p* < 0.001). In the cases' group, the median number of days with symptoms was 365 (interquartile range = 745). Only DASH score in the cases' group showed a normal distribution, thus non-parametric statistics were applied in all other situations.

**Table 1:** Socio-demographic and clinical characteristics of all participants.

**Characteristics**	**Cases**	**Controls**	***p*-value**
Number of participants	140	62	
Sex, women, *n* (%)	108 (77)	47 (76)	0.86
Age, years, median [IQR]^*^	56 [16]	56 [17]	0.54
Exam on dominant side, *n* (%)	89 (64)	38 (61)	0.76
Work status and occupation, *n* (%)			0.43
Professionals^†^	17 (12)	13 (21)	
Technicians and associate professionals	19 (14)	12 (19)	
Services and sales workers	29 (21)	9 (15)	
Other professions	29 (21)	9 (15)	
Home duties	12 (9)	5 (8)	
Unemployed or retired	34 (24)	14 (23)	
Previous shoulder tendinopathy, *n* (%)	34 (24)	3 (5)	**0.001**
Diagnosis of previous rotator cuff tear, *n* (%)	5 (4)	0	0.33
Department of origin, *n* (%)			**0.001**
Orthopedics	64 (46)	23 (37)	
Physical medicine and rehabilitation	32 (23)	11 (18)	
Neurosurgery	18 (13)	1 (2)	
Other	26 (19)	27 (44)	
DASH score, median [IQR]^*^	54 [25]	0 [3]	**<0.001**
Positive shoulder tests, *n* (%)			
Jobe	103/132 (78)	2 (3)	**<0.001**
Neer	73/135 (54)	0	**<0.001**
Palm-up	86/135 (64)	1 (2)	**<0.001**
Active range of motion, total degrees lost,	30 [140]	0 [20]	**<0.001**
median [IQR]^*^			

*Unless otherwise indicated, data are numerators and data in parentheses are percentages, compared using Fisher's exact test; denominator was omitted when equal to the number of participants in first row. IQR, interquartile range; DASH, disabilities of the arm, shoulder, and hand outcome measure. Statistically significant p-values are highlighted in bold*.

* *Data are median and data in brackets are interquartile range, compared using the Mann-Whitney U-test*.

† *Includes engineers, medical doctors, teachers, lawyers*.

**Figure 2:**
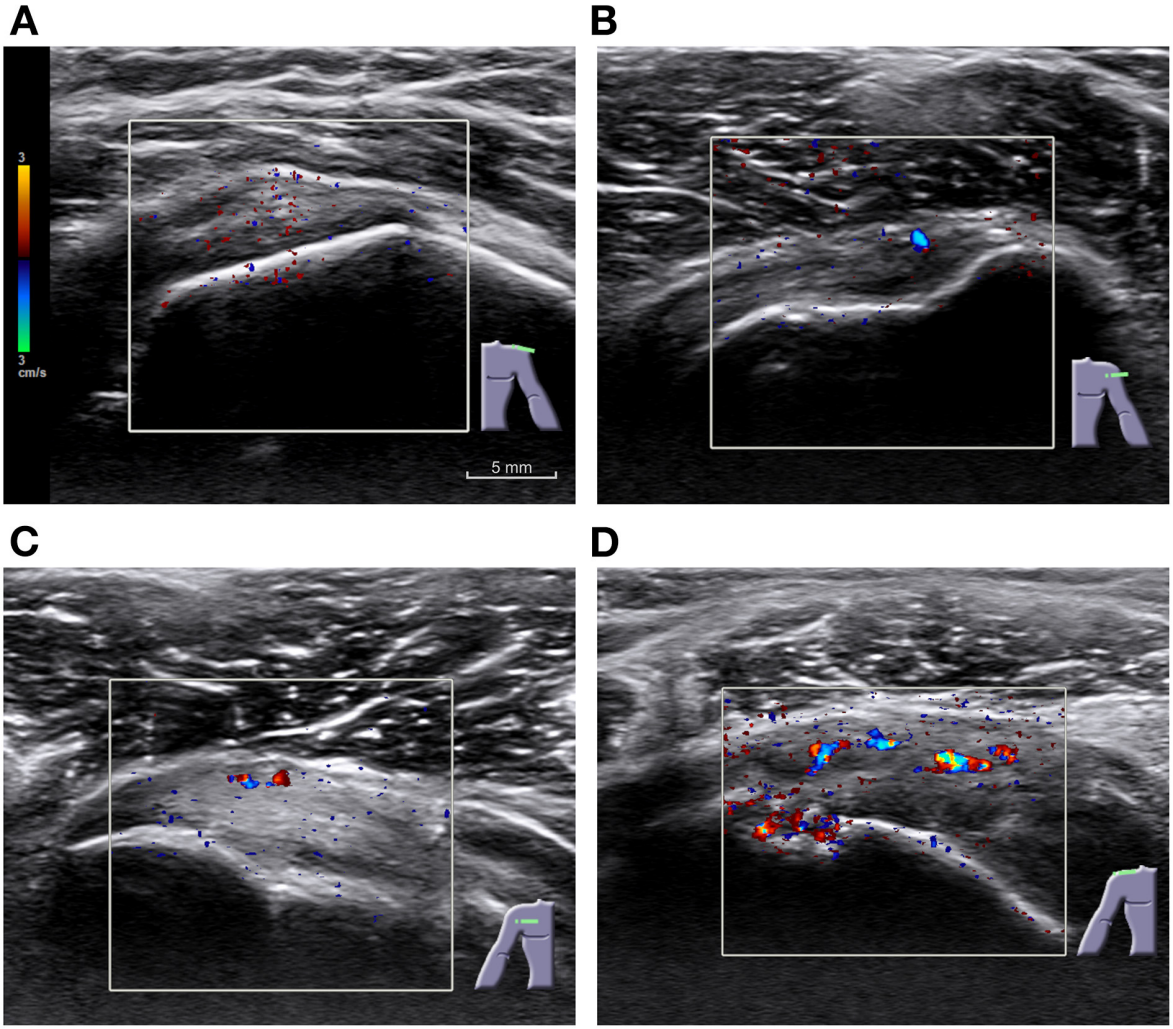
Examples of different grades of Color Doppler in rotator cuff tendons of symptomatic participants. **(A)** Grade 0, with no significative signal and only minimal scattered noise; **(B)** grade 1, with one or two points or short lines; **(C)** grade 2, showing three to six points or short lines; and **(D)** grade 3, with more than six points, a continuous line, or a bar. A pulse repetition frequency (PRF) of 488 Hz was used. Scale and color coding are identical across images.

A comparison of condensed US findings is shown in Table 2. Doppler grades (illustrated in Figure 2), subacromial effusion, number of calcifications, tendon tear, echotexture change and insertional thickening, humeral tuberosity cortical irregularity, and interruption of abduction revealed differences between cases and controls. The dimension of calcifications and their distance to tendon insertion deserved detailed assessment. The number of tendons with moderate echotexture changes, effusion thickness, and Doppler grades were low, even in the cases' group.

**Table 2:** Ultrasound findings comparation between cases and controls.

**Findings**	**Cases**	**Controls**	***p*-value**
	**(*n* = 140)**	**(*n* = 62)**	
Number of calcifications	1.5 [3]	1.0 [2]	**0.02**
Diameter of largest calcification, mm, median [IQR]	2.7 [5.2]	1.5 [4.0]	0.06
Largest distance of calcification to insertion, mm, median [IQR]	1.9 [3.2]	1.0 [2.8]	0.08
Number of tendons with mild heterogeneity, median [IQR]	1.0 [1]	1.0 [1]	0.61
Number of tendons with moderate heterogeneity, median [IQR]	0.5 [1]	0.0 [0]	**<0.001**
Number of tendons with mild hypoechogenicity, median [IQR]	1.0 [2]	1.0 [1]	0.94
Number of tendons with moderate hypoechogenicity, median [IQR]	0.0 [1]	0.0 [0]	**<0.001**
Any tendon insertional thickening, *n* (%)^*^	47 (34)	12 (19)	**0.045**
Humeral tuberosity cortical irregularity, *n* (%)^*^	81 (58)	23 (37)	**0.009**
Rotator cuff tear, *n* (%)^*^	29 (21)	2 (3)	**0.001**
Full thickness tear, *n* (%)^*^	7 (5)	1 (2)	0.44
Partial thickness tear, *n* (%)^*^	22 (16)	1 (2)	**0.003**
Subacromial conflict signs			
Interruption of abduction, *n* (%)^*^	12 (9)	0	**0.02**
Pooling of liquid on abduction, *n* (%)^*^	2 (1)	0	1.00
CAL displacement increment, mm, median [IQR]	1.3 [1.8]	1.1 [1.5]	0.22
Supraspinatus thickness, mm, median [IQR]	5.6 [1.4]	5.6 [1.0]	0.71
Glenohumeral effusion, mm, median [IQR]	0.0 [0.0]	0.0 [1.0]	0.14
Subacromial effusion, mm, median [IQR]	0.0 [0.0]	0.0 [0.0]	**0.004**
Acromioclavicular capsule distension, mm, median [IQR]	0.0 [0.0]	0.0 [0.0]	0.17
Acromioclavicular osteoarthritis, *n* (%)^*^	35 (25)	14 (23)	0.86
Synovial thickening, *n* (%)^*^	3 (2)	0	0.55
Long head of biceps tendon lesion, *n* (%)^*^	4 (3)	1 (2)	1.00
Color Doppler grade, median [IQR]	0.0 [0]	0.0 [0]	**0.002**
Power Doppler grade, median [IQR]	0.0 [0]	0.0 [0]	**0.004**
Doppler signal within calcification, *n* (%)^*^	2 (1)	0	1.00
Doppler signal near calcifications, *n* (%)^*^	7 (5)	0	0.10
Doppler twinkling sign, *n* (%)^*^	5 (4)	1 (2)	0.67

*Unless otherwise indicated, data are median and data in brackets are interquartile range, compared using the Mann-Whitney U-test. IQR, interquartile range; CAL, coracoacromial ligament. Statistically significant p-values are highlighted in bold*.

* *Data are numerators and data in parentheses are percentages, compared using Fisher's exact test; the denominator used to calculate the percentages was the number of participants in column heading*.

A total of 405 calcifications were found, from which 377 were described (Table 3). Calcification diameter ranged from 0.5 to 16.7 mm.

**Table 3:** Characteristics of individual calcifications.

**Characteristics**	**Cases**	**Controls**	***p*-value**
Total number of calcifications described	291	86	
Diameter, greater axis in mm, median [IQR]^*^	2.4 [2.5]	2.9 [2.7]	0.07
Distance to insertion, mm, median [IQR]^*^	1.8 [1.6]	1.9 [1.6]	0.13
Location, *n* (%)			0.23
Supraspinatus	86 (30)	30 (35)	
Infraspinatus	65 (22)	26 (30)	
Subscapularis	106 (36)	26 (30)	
Teres minor	25 (9)	4 (5)	
Supraspinatus/Infraspinatus	8 (3)	0	
Infraspinatus/Teres minor	1 (0)	0	
Geometry, *n* (%)			0.56
Linear	24 (8)	10 (12)	
Oval	155 (53)	42 (49)	
Polyhedral	112 (38)	34 (40)	
Irregular shape, *n* (%)	124 (43)	29 (34)	0.17
Poorly defined shape, *n* (%)	42 (14)	15 (17)	0.50
Acoustic shadow, *n* (%)			0.69
Absent	258 (89)	79 (92)	
Mild	29 (10)	7 (8)	
Strong	4 (1)	0	
Grouped, *n* (%)	37 (13)	9 (10)	0.71

*Unless otherwise indicated, data are numerators and data in parentheses are percentages, compared using Fisher's exact test; the denominator used to calculate the percentages was the number of calcifications in first row. IQR, interquartile range*.

* *Data are median and data in brackets are interquartile range, compared using the Mann-Whitney U-test*.

### Subgroups and Cutoff Analysis

The frequencies of positive findings by age subgroup, with relevant cutoffs applied, are outlined in Table 4.

**Table 4:** Comparation between cases and controls by cutoff levels and age group.

**Findings**	**Cases**	**Controls**	***p*-value**
	***n* (%)**	***n* (%)**	
Number of participants	140	62	
<56 years old	67	31	
≥56 years old	73	31	
Number of calcifications			
≥1	103 (74)	38 (61)	0.10
≥3	54 (39)	14 (23)	0.04
≥4	30 (21)	6 (10)	**0.048**
≥6	14 (10)	1 (2)	**0.04**
Diameter of largest calcification, mm			
≥6 mm	26 (19)	8 (13)	0.42
<56 years old	11 (16)	0	**0.02**
≥56 years old	15 (21)	8 (26)	0.61
Largest distance of calcification to insertion, mm			
≥5 mm	22 (16)	3 (5)	**0.04**
≥6 mm	15 (11)	1 (2)	**0.03**
<56 years old	2 (3)	1 (3)	1.00
≥56 years old	13 (18)	0	**0.009**
At least one RC tendon with moderate heterogeneity	63 (45)	9 (15)	**<0.001**
At least one RC tendon with moderate hypoechogenicity	66 (47)	8 (13)	**<0.001**
Any tendon insertional thickening	47 (34)	12 (19)	**0.045**
Specific tendon insertional thickening			
Supraspinatus	17 (12)	0	**0.002**
Infraspinatus	27 (19)	9 (15)	0.55
Subscapularis	10 (7)	0 (0)	**0.03**
Humeral tuberosity cortical irregularity	81 (58)	23 (37)	**0.009**
Specific location of cortical irregularity			
Supraspinatus insertion	32 (23)	6 (10)	**0.03**
Infraspinatus insertion	30 (21)	11 (18)	0.71
Subscapularis insertion	42 (30)	9 (15)	**0.02**
Rotator cuff tear	29 (21)	2 (3)	**0.001**
Any subacromial effusion	17 (12)	0	**0.002**
Color Doppler grade > 0 in RC tendons	26 (19)	2 (3)	**0.003**
Power Doppler grade > 0 in RC tendons	29 (21)	3 (5)	**0.003**

*Data are numerators and data in parentheses are percentages, compared using Fisher's exact test; the denominator used to calculate the percentages was the adequate number of participants in first rows. RC, rotator cuff. Statistically significant p-values are highlighted in bold*.

The presence of calcifications (in any number) did not differ between groups, but three or more calcifications were found more frequently in participants with symptoms. In the younger subgroup of participants (<56 years old), a calcification diameter ≥6 mm was only found in the presence of symptoms. Distance to insertion also showed a higher difference between groups when a 6 mm cutoff was applied, only in older participants, with no difference in the younger subgroup.

Echotexture moderate changes, of at least one RC tendon, were more frequent in cases than in controls, for all ages (with *p* < 0.001, both for heterogeneity and hypoechogenicity). Some specific tendon echotexture changes also appeared more frequently in the cases' group, but with a lower significance. Insertional thickening of at least one tendon was more common in cases than in controls, but a greater difference was found when isolated supraspinatus and subscapularis were assessed. Infraspinatus thickening was not proved to be different between cases and controls. Also of interest, humeral tuberosity cortical irregularity at the supraspinatus and subscapularis insertion sites was more associated with cases than controls, which was not the case for the infraspinatus insertion.

Rotator cuff tears were more frequent in the cases' group, for all ages. Supraspinatus tendon was the site for 7 of 11 full thickness tears and 22 of 27 partial thickness tears. Any fluid on subacromial bursa (minimum registered positive value was 0.7 mm) was only found in symptomatic participants.

The presence of any sign in both Color and Power Doppler was more frequent in participants with symptoms, with few controls displaying any signal. Doppler predominant locations were supraspinatus tendon (found in 10% of the symptomatic participants) and subscapularis tendon (8.6%). Twinkling artifact was found in 5 of 26 cases and in 1 of 2 controls with color Doppler signal.

### Interactions and Reliability

The older subgroup (≥56 years old) had a higher calcification number, diameter, and distance to insertion (*p* < 0.001, Mann–Whitney *U*-test) and more frequent partial thickness tears and humeral tuberosity cortical irregularities (respectively, *p* = 0.002 and *p* = 0.005, Fisher's exact test). No differences between sexes were found in US relevant findings. A positive color Doppler grade was more frequent on the dominant side (26/127, 20.5%) than on the non-dominant side (4/75, 5.3%), *p* = 0.004.

Color and Power Doppler grades were found to be strongly associated, with a Spearman correlation coefficient of 0.917.

The analysis of DASH scores in the cases' group depicted higher values (independent *t*-test) in participants with moderate heterogeneity in at least a tendon (*M* = 57 vs. *M* = 49, *p* = 0.01) and with moderate hypoechogenicity in at least a tendon (*M* = 57 vs. *M* = 49, *p* = 0.02). The presence of other US findings did not result in significantly different DASH scores, in the cases' group.

For the presence of humeral tuberosity cortical irregularity, inter-observer agreement was substantial (33), *k* = 0.72 (0.36–1.0). For the diameter of largest calcification, ICC = 0.98 (0.93–0.99) was obtained, and an ICC = 0.90 (0.72–0.96) was found for the largest distance from calcification to insertion. Reliability analysis for other less frequent findings, including Doppler, resulted in wider, not trustable, CIs.

### Classification Algorithm

**Figure 3:**
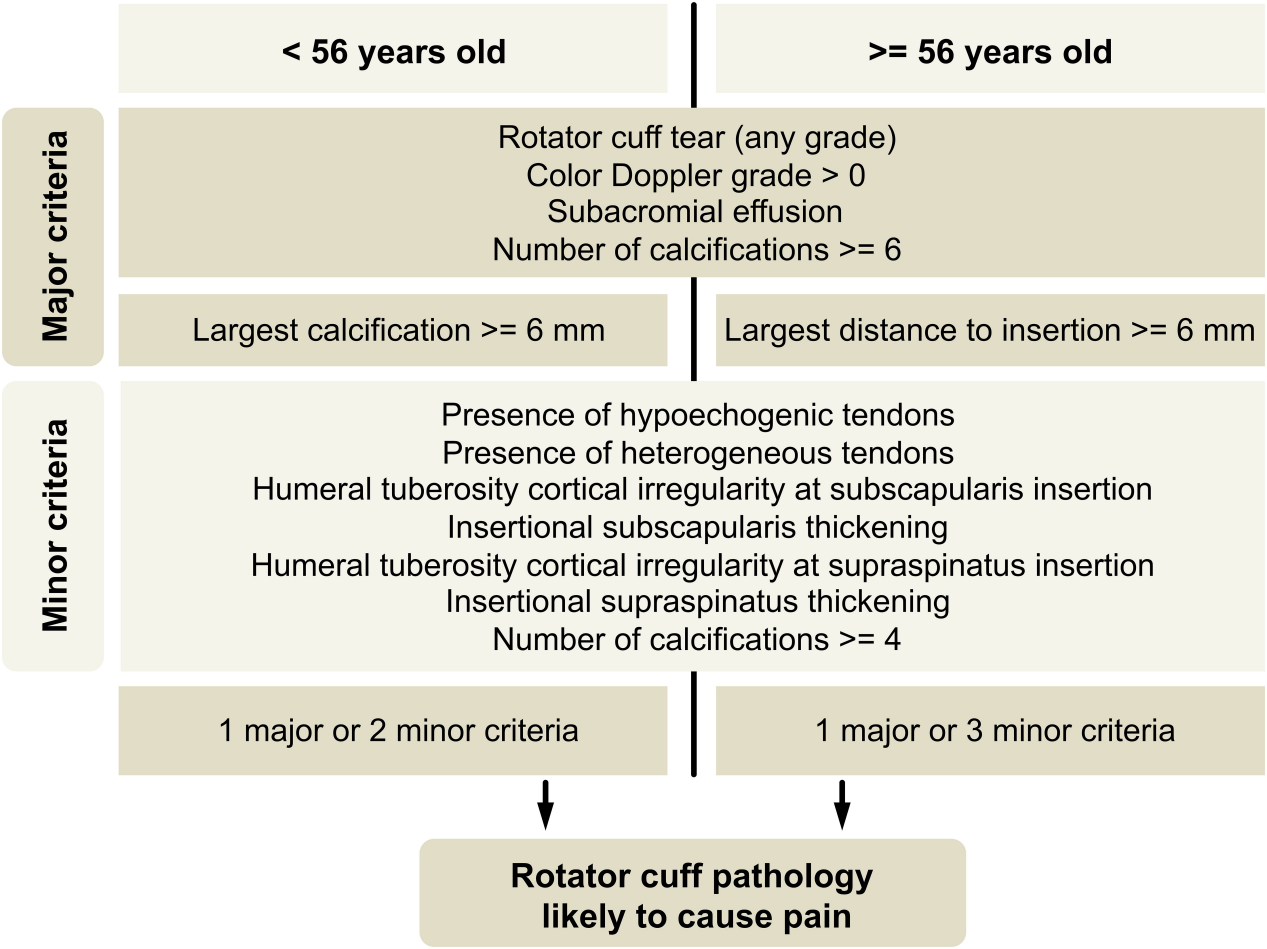
Classification algorithm proposal. Cortical irregularity and insertional thickening related to subscapularis and supraspinatus tendons should be taken as separate criteria. Mild, questionable, heterogeneity, or hypoechogenicity of tendons should not be considered.

**Figure 4:**
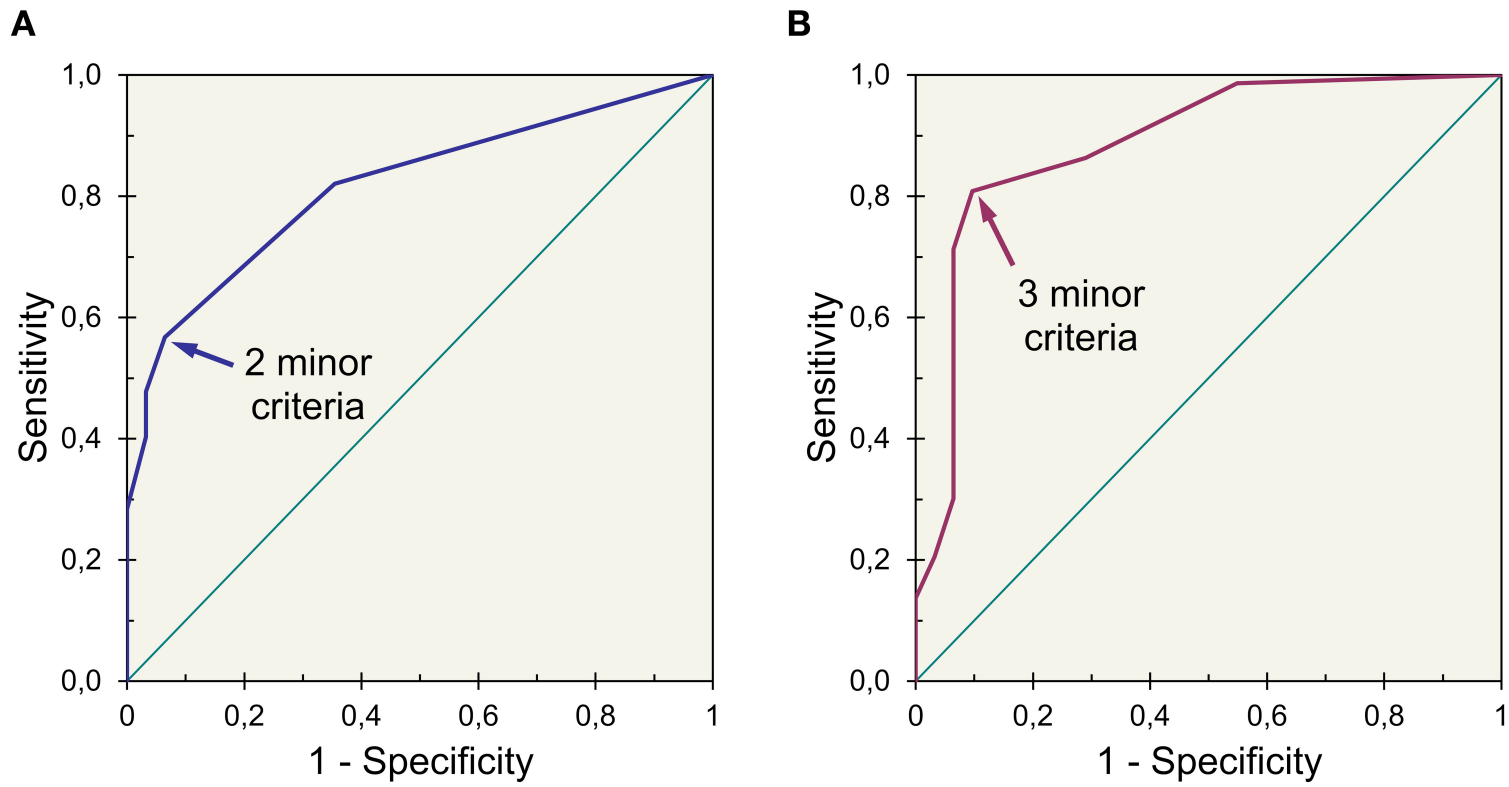
Receiver operating characteristic curves of an index obtained for **(A)** younger (<56 years) and **(B)** older (≥56 years) participants showing different thresholds for best accuracy (index established by adding three points for major criteria and one point for minor criteria).

An algorithm proposal aiming for high specificity, constructed with US findings as major or minor criteria, is showed (Figure 3). Major criteria were meant to provide a positive result alone. The best threshold for the number of minor criteria required, estimated by analysis of ROC curves, was two minor criteria for participants with <56 years and three minor criteria for participants with 56 years or older (Figure 4).

This algorithm provides a classification, in this study sample, with a sensitivity of 69%, 97/140 (95% CI: 61–77%) and a specificity of 92%, 57/62 (95% CI: 85– 99%). If minor criteria based on tendon echotexture changes and insertional thickening are withdrawn, an algorithm with a sensitivity of 59%, 83/140 (95% CI: 51–67%) and a specificity of 94%, 58/62 (95% CI: 88–99%), will result.

## Discussion

Although US findings associated with shoulder pain have been described, substantial overlap exists between painful and asymptomatic shoulders. There are also some discrepancies between studies with different designs. In this study, using calculated thresholds and taking age into account by analysis of two similar size subgroups, it was possible to find differences in painful shoulders concerning calcification size (*p* = 0.02, age < 56 years) and the presence of color Doppler (*p* = 0.003), and also regarding calcification distance to insertion, number of calcifications, tendon echotexture and insertional thickening, osseous irregularity, and the presence of RC tear or subacromial effusion. An algorithm based on US findings providing a strong association with the presence of symptoms, with 57/62 (92%) specificity (on the study sample), was constructed.

Our results confirmed and extended the findings of previous reports. On their study, including only participants with proved calcified deposits (13), Le Goff et al. reported a relationship between the presence of symptoms and the size of calcifications, although they did not look for a cutoff. They found thickening of the subacromial bursa to be frequent in symptomatic shoulders, as reported by Aina et al. (34) and in accordance with our results. They also, as described by Chiou et al. (17), found Doppler within or near calcifications to be strongly associated with symptoms. We also found the presence of any grade of color Doppler, related or not to calcifications, to be predictive of symptoms. The PRF that we have used was low, raising concern about a possible overvaluation of the Doppler signal. However, the number of controls with Doppler signal (false positives) was only 3%. We found evidence of twinkling artifact, which could be misleading, in only 5 of the 26 cases with color Doppler signal.

In their US study on a cohort of women (14), Sansone et al. found calcium deposits to be more frequent than previously reported in radiographic observations, and that calcifications, besides supraspinatus, were also frequent in infraspinatus and subscapularis. No relationship between calcifications' size and pain was confirmed by that study, which classified dimensions in only three classes and did not consider the influence of age. We found even more frequent tendon calcifications, present in more than half of the participants, both with and without symptoms, perhaps due to the older age of our sample. The distribution of calcific deposits was comparable among those three tendons and teres minor also showed a non-negligible number of calcifications. In our sample, a higher number of calcifications was related to pain, but not the sole presence of one calcification. We found no association between the presence of pain and calcifications shape or acoustic shadow.

The relevance of the distance from calcification to tendon insertion was not, to our knowledge, previously reported on US. It suggests that, in older people, calcifications located closer to insertion are not necessarily pathological. The greater relevance of calcification size in a younger age subgroup indicates that degenerative changes are less important at this stage, and a different process, such as calcifying tendinitis, can prevail earlier, as hypothesized by Uhtoff et al. (11).

As expected, RC tears were associated with pain, as well as humeral tuberosities irregularities (other than the more common irregularity at insertion of infraspinatus), previously described to be associated with tears (35). Glenohumeral effusion and signs of acromioclavicular osteoarthritis or subacromial conflict were not associated with pain.

The predominance of Doppler in the dominant side suggests that movement or load have a role in the development of vascularization in painful shoulders.

Age, considered an important factor in the development of calcifications, was similar between groups (cases and controls), as a result of the matching process on control selection. This allowed to exclude an effect of age on the case/control comparisons. Likewise, gender and side dominancy distribution were similar between groups.

Our study had several limitations. Shoulder tendinopathy is a relapsing condition; therefore, a history of previous shoulder tendinopathy was more frequent in the cases' group. Thus, the US changes were also due, at least partly, to prior episodes. Professions with potentially less shoulder effort were slightly more frequent in controls, but this difference was not statistically significant. The radiologist who performed the US exams was not fully blinded to the condition of each participant being a case or a control; this limitation is hard to be overcome, as US is a dynamic exam and patients will spontaneously reveal pain on movement. Algorithm minor criteria relying on tendon echotexture changes and thickening are subjective and should be used with caution, as rating could be unreliable; if those criteria are not used, a slightly better specificity and a reduced sensitivity will result. Future work could establish the reliability of Color Doppler grading and tendon changes rating. This classification algorithm will need to be validated in an independent population.

The results here reported cannot be applied to patients with chronic inflammatory diseases, including rheumatic diseases, as they were not included in this study as they tend to show different US characteristics. Indeed, US and Power Doppler in rheumatoid arthritis with symptomatic shoulder frequently shows glenohumeral, subdeltoid bursa, and biceps tendon sheath effusion and/or synovitis and can also display erosions and degenerative changes like osteophytes and RC tear and calcification (36). Regarding polymyalgia rheumatica, subdeltoid bursitis, biceps tenosynovitis, or glenohumeral synovitis are prominent features, and according to the 2012 EULAR/ACR collaborative initiative (37) these findings are useful in discriminating polymyalgia rheumatica from other shoulder conditions (although less useful in discriminating from rheumatoid arthritis).

We hope this work and the US criteria here described will help, when symptoms and physical examination are ambiguous, the diagnostic differentiation between RC/subacromial pathology and other local conditions or referred pain.

In conclusion, calcifications with a diameter of 6 mm or more, in patients younger than 56 years old, or with a distance from tendon insertion of 6 mm or more, in older patients, are associated with pain. The presence of any Doppler signal also is associated with shoulder complaints. An algorithm based on a set of specific, yet less frequent, US criteria, provides a strong association with the presence of symptoms.

## Data Availability Statement

The raw data supporting the conclusions of this article will be made available by the authors, without undue reservation.

## Ethics Statement

The studies involving human participants were reviewed and approved by Comissão de Ética do Centro Hospitalar Universitário Lisboa Norte (CHULN) e do Centro Académico de Medicina de Lisboa (CAML)—Approval Letter Ref. n. 413/17. The patients/participants provided their written informed consent to participate in this study.

## Author Contributions

JJ, SB, and PM: literature research. SB and PM: clinical studies. JJ: statistical analysis. JJ and SB: manuscript editing. All authors: study concepts and design, data acquisition, analysis and interpretation, manuscript drafting and revision for important intellectual content, approval of final version of submitted manuscript. All authors agree to be accountable for the content of the work.

## Conflict of Interest

The authors declare that the research was conducted in the absence of any commercial or financial relationships that could be construed as a potential conflict of interest.

## Publisher's Note

All claims expressed in this article are solely those of the authors and do not necessarily represent those of their affiliated organizations, or those of the publisher, the editors and the reviewers. Any product that may be evaluated in this article, or claim that may be made by its manufacturer, is not guaranteed or endorsed by the publisher.

## Supplementary Material

Supplementary Figure 1 Measurement of the distance from calcification to osseous insertion. The distance (arrow) was measured from the lateral limit of the calcification to the bone contour, in a direction parallel to the tendon fibers.

Supplementary Figure 2 Convexity of coracoacromial ligament (CAL). The displacement (arrow) was measured as the greatest distance from a line joining acromion and coracoid process to the ligament.

Supplementary Table 1 Comprehensive list of exclusion criteria.

Supplementary Table 2 US findings definitions.

Supplementary Table 3 Sensitivity and specificity of individual algorithm criteria.

## Abbreviations

US: ultrasound
RC: rotator cuff.
