# Supplementary figures and images for: Ultrasound Features Associated With Shoulder Complaints: Calcifications Larger Than 6 mm in Young Patients and Positive Doppler Are Associated With Pain
Source: Front Med (Lausanne). 2021 Nov 19;8:715423. doi: 10.3389/fmed.2021.715423 (PMC8639518; doi:10.3389/fmed.2021.715423)

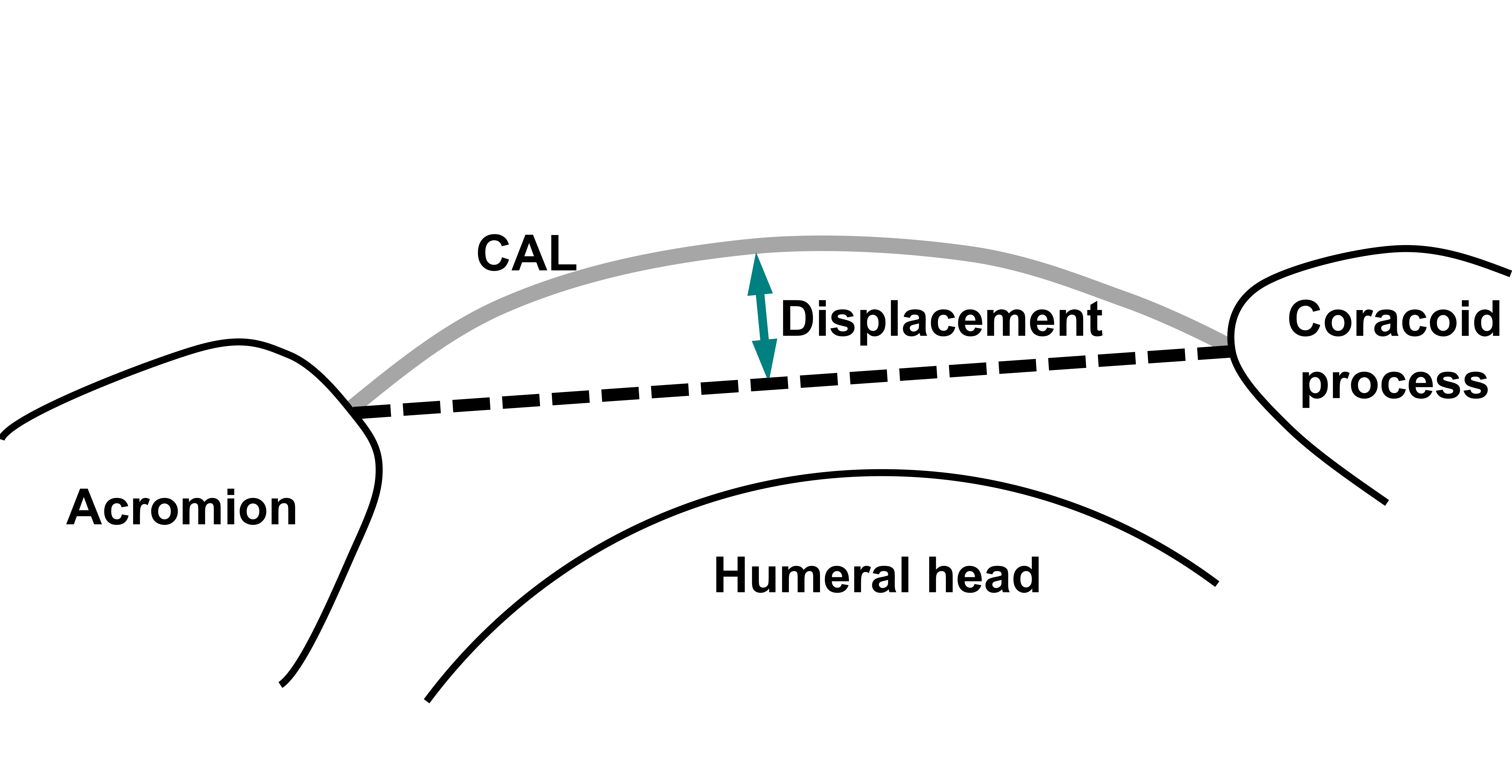

Supplement: Supplementary Figure 2 — Convexity of coracoacromial ligament (CAL). The displacement (arrow) was measured as the greatest distance from a line joining acromion and coracoid process to the ligament. [file Image_2.TIF]
