# Supplementary material for: Ultrasound Features Associated With Shoulder Complaints: Calcifications Larger Than 6 mm in Young Patients and Positive Doppler Are Associated With Pain
Source: Front Med (Lausanne). 2021 Nov 19;8:715423. doi: 10.3389/fmed.2021.715423 (PMC8639518; doi:10.3389/fmed.2021.715423)
Supplement: Supplementary Table 1 — Comprehensive list of exclusion criteria. [file Data_Sheet_1.docx]

Supplementary Material

# Supplementary Figures and Tables

## Supplementary Figure legends

**Figure S1.** Measurement of the distance from calcification to osseous insertion. The distance (arrow) was measured from the lateral limit of the calcification to the bone contour, in a direction parallel to the tendon fibers.

**Figure S2.** Convexity of coracoacromial ligament (CAL). The displacement (arrow) was measured as the greatest distance from a line joining acromion and coracoid process to the ligament.

# Supplementary Tables

Please find 3 supplementary tables on the next pages.

**Table S1.** Comprehensive list of exclusion criteria

| Criteria | Remarks |
| --- | --- |
| Previous shoulder surgery | On the examined shoulder |
| Recent (2 weeks) local invasive therapy * | On the examined shoulder |
| Chronic inflammatory disease | Including rheumatoid arthritis, systemic lupus erythematosus, psoriasis, gout |
| Oncologic disease | Active in the past 5 years |
| Chronic infection | Including tuberculosis, hepatitis, AIDS |
| Glenohumeral osteoarthritis | Previously known or found on US exam |
| Unable to participate | Minors, non-imputable or subjects unable to understand and accept informed consent or answer the study questionnaire |
| Exam for legal purposes | Requested in the setting of occupational medicine, by a court of law or intending to define compensation or absence from work |
| Pregnancy * | Pregnant women or who have given birth in the previous month |

AIDS - acquired immunodeficiency syndrome; US - ultrasound

* No participants were excluded by these criteria

**Table S2.** US findings definitions

| Findings | Definition / remarks |
| --- | --- |
| Calcification | A compatible echogenic image, identified in two orthogonal planes and not otherwise explained, with or without acoustic shadow |
| Number | Calcifications >= 0.5 mm were counted |
| Diameter | Greater diameter measured, in millimeters |
| Distance to insertion | Distance to osseous insertion in a direction parallel to tendon fibers, in millimeters (Fig S1) |
| Location | Tendon(s) or structure(s) where calcification was found |
| Shape geometry | Oval - formed by arc segments; Polyhedral - with multiple faces; Linear - great axis exceeded 6 times short axis |
| Shape regularity | Irregular – with depressions and prominences at uneven spaces |
| Shape definition | Poorly defined - without a clear definition of contour |
| Acoustic shadow | Absent, Mild or Strong |
| Grouping | Grouped - part of a group of 3 or more calcifications in a radius of 3 millimeters |
| Tendon echotexture changes |  |
| Grade | Mild - possible change; Moderate - definite change |
| Heterogeneity | Inhomogeneity, variable echogenicity along the tendon |
| Hypoechogenicity | Compared to muscle |
| Tendon insertional thickening | Thickening of a tendon as it comes closer to osseous insertion |
| Humeral tuberosity irregularity | Osseous irregularity at the insertion of rotator cuff tendons |
| Rotator cuff tear signs | Non-visualization of a tendon; Presence of a cleft; Focal thinning; Loss of convexity; Focal hypoechogenicity |
| Rotator cuff tear grade | Full thickness or Partial thickness |
| Subacromial conflict signs |  |
| Interruption of abduction | Stop or hesitation on dynamic evaluation |
| Pooling of liquid | Lateral pooling of liquid on subacromial bursa during abduction |
| CAL displacement increment | Difference, in millimeters, from neutral to maximal convexity in dynamic evaluation with abduction and internal rotation (Figure S2) |
| Supraspinatus thickness | Measured in millimeters immediately lateral to acromion |
| Glenohumeral effusion | Thickness in millimeters |
| Subacromial effusion | Thickness in millimeters |
| AC capsule distension | Thickness in millimeters |
| Signs of osteoarthritis | AC or glenohumeral |
| Synovial thickening | Presence or absence |
| LHB tendon lesion | Non-visualization or subluxation |
| Color and Power Doppler grade | 0 - no signal; 1 - one or two points or short lines; 2 - three to six points or short lines; 3 - more than six points, continuous line, or bar |
| Doppler signal location | Within calcification; Near calcifications; Tendon(s) or structure(s) where Doppler signal was found |
| Doppler twinkling sign | Fast alternance between colors near the limit of spectrum |

Note. Any additional finding was recorded in free text. AC - acromioclavicular; CAL - coracoacromial ligament; LHB - long head of biceps; US - ultrasound

**Table S3.** Sensitivity and specificity of individual algorithm criteria

| Criteria | On sample | |
| --- | --- | --- |
|  | Sensitivity | Specificity |
| Major criteria |  |  |
| Rotator cuff tear (any grade) | 29/140 (21%) | 60/62 (97%) |
| Color Doppler grade > 0 | 26/140 (19%) | 60/62 (97%) |
| Diameter of largest calcification >= 6 millimeters  (< 56 years old) | 11/67 (16%) | 31/31 (100%) |
| Largest distance of calcification to insertion >= 6 millimeters  (>= 56 years old) | 13/73 (18%) | 31/31 (100%) |
| Subacromial effusion | 17/140 (12%) | 62/62 (100%) |
| Number of calcifications >= 6 | 14/140 (10%) | 61/62 (98%) |
| Minor criteria |  |  |
| Presence of at least one tendon with moderate hypoechogenicity | 66/140 (47%) | 54/62 (87%) |
| Presence of at least one tendon with moderate heterogeneity | 63/140 (45%) | 53/62 (85%) |
| Humeral tuberosity cortical irregularity at subscapularis insertion | 42/140 (30%) | 53/62 (85%) |
| Insertional subscapularis thickening | 10/140 (7%) | 62/62 (100%) |
| Humeral tuberosity cortical irregularity at supraspinatus insertion | 32/140 (23%) | 56/62 (90%) |
| Insertional supraspinatus thickening | 17/140 (12%) | 62/62 (100%) |
| Number of calcifications >= 4 | 30/140 (21%) | 56/62 (90%) |

Note. A positive classification in algorithm is indicated by the presence of 1 major criterion or by the conjunction of 2 (in subjects < 56 years) or 3 (>= 56 years) minor criteria.
